# Supplementary material for: Factors influencing infertile couples’ decisions to seek healthcare: A mixed methods study from Islamabad, Pakistan
Source: PLOS Glob Public Health. 2026 Apr 15;6(4):e0006275. doi: 10.1371/journal.pgph.0006275 (PMC13082636; doi:10.1371/journal.pgph.0006275)
Supplement: S1 Checklist — (DOCX) [file pgph.0006275.s001.docx]

# STROBE Checklist for Cross-Sectional Study

Study Title: Factors Influencing Infertile Couples' Decisions to Seek Healthcare: A Mixed Methods Study from Islamabad, Pakistan

| Section | Item No | Recommendation | Addressed in Paper (with Page Reference) |
| --- | --- | --- | --- |
| Title and Abstract | 1(a) | Indicate study design in title/abstract | ✔ Abstract (Page 1): Described as 'mixed-methods' and 'cross-sectional' |
|  | 1(b) | Informative, balanced abstract summary | ✔ Page 1 |
| Introduction | 2 | Scientific background & rationale | ✔ Pages 2–4 |
|  | 3 | Specific objectives & hypotheses | ✔ Page 5 |
| Methods | 4 | Key elements of study design | ✔ Page 6 |
|  | 5 | Setting, locations, dates of recruitment/data collection | ✔ Page 6 |
|  | 6(a) | Eligibility criteria and selection methods | ✔ Pages 6–7 |
|  | 7 | Variables defined (outcomes, exposures, confounders) | ✔ Pages 6–8 |
|  | 8 | Data sources/measurement methods | ✔ Pages 7–8 |
|  | 9 | Efforts to address potential bias | ✔ Page 8 |
|  | 10 | Study size explanation | ✔ Page 6 |
|  | 11 | Handling of quantitative variables | ✔ Page 8 |
|  | 12(a) | Statistical methods including confounder control | ✔ Page 8 |
|  | 12(b) | Subgroups/interactions | ✔ Page 8 |
|  | 12(c) | Missing data explanation | ❌ None |
|  | 12(d) | Sampling strategy in analysis | ✔ Page 8 |
|  | 12(e) | Sensitivity analyses | ❌ None |
| Results | 13(a) | Participant numbers at each study stage | ✔ Page 6, 9 |
|  | 13(b) | Reasons for non-participation | ✔ Page 7 |
|  | 13(c) | Flow diagram | ❌ Not included |
|  | 14(a) | Characteristics of study participants | ✔ Page 9 |
|  | 14(b) | Missing data per variable | ❌ N/A |
|  | 15 | Outcome events or summary measures | ✔ Pages 9–11 |
|  | 16(a) | Unadjusted & adjusted estimates with CIs | ✔ Page 11–12 |
|  | 16(b) | Category boundaries of variables | ✔ Page 10–11 |
|  | 16(c) | Relative/absolute risk translation | ❌ N/A |
|  | 17 | Other analyses (subgroups/sensitivity) | ✔ Page 11–12 |
| Discussion | 18 | Key results with respect to objectives | ✔ Page 13 |
|  | 19 | Study limitations (bias/imprecision) | ✔ Page 15 |
|  | 20 | Interpretation considering evidence & limitations | ✔ Page 13–14 |
|  | 21 | Generalisability/external validity | ✔ Page 14–15 |
| Other Information | 22 | Funding & role of funders | ✔ Page 16 |
