# Supplementary material for: Factors influencing infertile couples’ decisions to seek healthcare: A mixed methods study from Islamabad, Pakistan
Source: PLOS Glob Public Health. 2026 Apr 15;6(4):e0006275. doi: 10.1371/journal.pgph.0006275 (PMC13082636; doi:10.1371/journal.pgph.0006275)
Supplement: S1 File — (DOCX) [file pgph.0006275.s002.docx]

**Questionnaire**

**Socio Demographic Information**

Age:

1. ≤19
2. 20-29
3. 30-34
4. 35-39
5. 40-49

Occupation

1. Government employee
2. Private employee
3. Self employed
4. Unemployed/housewives

Type of family

1. Nuclear family
2. Joint family

Gender:

1) Male

2) Female

Educational level:

1) Primary

2) Secondary

3) Bachelor's degree

4) none

Socioeconomic status:

1) Low class

2) Middle class

3) High class

**Clinical profile**

Type of infertility.

1) Primary

2) Secondary

Duration of marriage:

1) Less than 1 year

2) 1-5 years

3) 6-10 years

4) More than 10 years

How long have you and your partner been trying to conceive?

1) Less than 1 year

2) 1-3 years

3) 4-6 years

4) More than 6 years

Have you sought medical advice for infertility before?

1) Yes

2) No

Time between marriage and treatment (years)

1. <1
2. 1-3
3. >3

**Care-Seeking practices:**

Who was the first person you consulted regarding your fertility concerns?

1) General practitioner/primary care physician

2) Obstetrician/gynecologist (OB/GYN)

3) Fertility specialist/reproductive endocrinologist

4) Friends/family members

What factors influenced your decision to seek medical advice for infertility?

1) Concern about age-related fertility decline

2) Unable to conceive after trying for a certain period

3) Recommendation from a healthcare professional

4) Others (spiritual, dai, homeopathy etc.)

Did you encounter any barriers or challenges while seeking infertility treatment?

1) Financial constraints

2) Lack of access to specialized services

3) Social stigma surrounding infertility

4) Family pressure

Treatment seeking behavior Advised by*

1. Self-seeking by couple
2. Household members
3. Friends

Decision to seek care taken by*

1. Wife
2. Husband
3. Both
4. Parents

Place of last treatment

1. private doctor/NGO/trust
2. govt. doctor
3. dai/midwife
4. none

Number of consultations

1. One-two
2. Three-four
3. Four-five
4. Six (or more)

**Social Support and Coping Mechanisms**

To what extent do you feel supported by your partner in dealing with infertility?

1) Strongly supported

2) Somewhat supported

3) Neutral

4) Not supported

Have you sought support from friends or family members regarding your infertility struggles?

1) Yes

2) No

Have you utilized any coping mechanisms to deal with the emotional stress of infertility? (e.g., support groups, therapy)

a. Yes

b. No

**Decision-making and Future Plans**

How do you and your partner make decisions regarding infertility treatment options?

1) Jointly, with mutual agreement

2) Mostly partner-driven

3) Mostly self-driven

**Additional Comments**

Is there anything else you would like to share about your experience with infertility and seeking care?
