# Supplementary material for: Factors influencing infertile couples’ decisions to seek healthcare: A mixed methods study from Islamabad, Pakistan
Source: PLOS Glob Public Health. 2026 Apr 15;6(4):e0006275. doi: 10.1371/journal.pgph.0006275 (PMC13082636; doi:10.1371/journal.pgph.0006275)
Supplement: Attachment — Submitted filename: Comments for Manuscript Number PGPH-D-25-02139.docx [file pgph.0006275.s003.docx]

**Reviewer Recommendation and Comments for Manuscript Number PGPH-D-25-02139**

Congratulations to the authors on the completion of the study and on putting together a good report on a relevant subject.

However, I have a few comments.

| s/n | Section | Comments | Recommendations |
| --- | --- | --- | --- |
| 1 | Data Collection, Management and Analysis | Sampling methods seem unclear; was it systematic random as stated here or purposive sampling as stated in the limitations | Consider reviewing and harmonizing these |
| 2 |  | There is a repetition of the inclusion criteria in the last paragraph with an additional information about use of contraception that was not part of the initial list. | Consider harmonizing these and avoid repetitions |
| 3 | Quantitative findings | The second sentence seeks to report the “average age” but a range was stated. | Kindly state the mean (with SD) |
| 4 |  | The 5^th^ sentence (Most had been married…) is unclear and has mathematical error; 139 of 269 is not 44.6%. | Consider recalculating and rewording this. |
| 5 | Table 1 | Under the ‘Treatment advised by’, what does ‘None’ represent? Missing data? Considering self-seeking is a category here. | Kindly provide clarifications |
| 6 | Table 2 | It is unclear how you have two categories of ‘Decision to seek medical care for infertility’ as Yes and No. Giving 100% of the participants already sought medical care.  More so, one cannot trace where the 175 participants under ‘Yes’ had come from. | Kindly review and provide clarifications |
| 7 |  | Similarly, there are 81 participants who were advised by family and friends on table 1 but a total of 87 on table 2 | Kindly review and rework |
| 8 | Discussion | Factors not adequately discussed | Authors should do more in highlighting the factors identified from the study and discussing the logistic regression findings after due diligence with the analyses. |
| 9 |  | The sentences after the citation 29 and 30 seem unfit for that section. | Consider moving those to the conclusion. |
| 10 | General comment | The use of medical ‘advice’ and medical ‘treatment’ interchangeably | These two are not technically the same and authors should review and decide which they are referring to. |
