# Supplementary material for: Factors influencing infertile couples’ decisions to seek healthcare: A mixed methods study from Islamabad, Pakistan
Source: PLOS Glob Public Health. 2026 Apr 15;6(4):e0006275. doi: 10.1371/journal.pgph.0006275 (PMC13082636; doi:10.1371/journal.pgph.0006275)
Supplement: Attachment — Submitted filename: Response to reviewers .docx [file pgph.0006275.s005.docx]

1. **Please ensure that your Ethics Statement is available in its entirety at the beginning of your Methods section, under a subheading 'Ethics Statement'.**

Response: Thank you for the feedback the ethic ctatement os now at the beginning of the method section

2. Please upload separate figure files in .tif or .eps format. Also, remove the figures from your manuscript file but keep the legends.

Response: we have made this change

3. We have noticed that you have uploaded Supporting Information files, but you have not included a list of legends. Please add a full list of legends for your Supporting Information files after the references list.

Response: we have made this change

4. We note that your Data Availability Statement is currently as follows: “all the data is already shared in the results section”
 
 Please confirm at this time whether or not your submission contains all raw data required to replicate the results of your study. Authors must share the “minimal data set” for their submission. PLOS defines the minimal data set to consist of the data required to replicate all study findings reported in the article, as well as related metadata and methods (https://journals.plos.org/plosone/s/data-availability#loc-minimal-data-set-definition).
 
 For example, authors should submit the following data:
 
 - The values behind the means, standard deviations and other measures reported;
 - The values used to build graphs;
 - The points extracted from images for analysis.
 
 Authors do not need to submit their entire data set if only a portion of the data was used in the reported study.
 
 If your submission does not contain these data, please either upload them as Supporting Information files or deposit them to a stable, public repository and provide us with the relevant URLs, DOIs, or accession numbers. For a list of recommended repositories, please see https://journals.plos.org/plosone/s/recommended-repositories.
 
 If there are ethical or legal restrictions on sharing a de-identified data set, please explain them in detail (e.g., data contain potentially sensitive information, data are owned by a third-party organization, etc.) and who has imposed them (e.g., an ethics committee). Please also provide contact information for a data access committee, ethics committee, or other institutional body to which data requests may be sent. If data are owned by a third party, please indicate how others may request data access.

If the reviewer comments include a recommendation to cite specific previously published works, please review and evaluate these publications to determine whether they are relevant and should be cited. There is no requirement to cite these works unless the editor has indicated otherwise.

Response: The data is present in the analysis. However all can be access on request to the first author by email.

Reviewers' comments:

Reviewer's Responses to Questions

**Comments to the Author**

1. Does this manuscript meet PLOS Global Public Health’s [publication criteria](https://journals.plos.org/globalpublichealth/s/criteria-for-publication)? Is the manuscript technically sound, and do the data support the conclusions? The manuscript must describe methodologically and ethically rigorous research with conclusions that are appropriately drawn based on the data presented.

Reviewer #1: Yes

Reviewer #2: Partly

Reviewer #3: Partly

2. Has the statistical analysis been performed appropriately and rigorously?

Reviewer #1: Yes

Reviewer #2: Yes

Reviewer #3: No

3. Have the authors made all data underlying the findings in their manuscript fully available (please refer to the Data Availability Statement at the start of the manuscript PDF file)?

The [PLOS Data policy](https://journals.plos.org/globalpublichealth/s/data-availability) requires authors to make all data underlying the findings described in their manuscript fully available without restriction, with rare exception. The data should be provided as part of the manuscript or its supporting information, or deposited to a public repository. For example, in addition to summary statistics, the data points behind means, medians and variance measures should be available. If there are restrictions on publicly sharing data—e.g. participant privacy or use of data from a third party—those must be specified.

Reviewer #1: Yes

Reviewer #2: Yes

Reviewer #3: No

4. Is the manuscript presented in an intelligible fashion and written in standard English?

PLOS Global Public Health does not copyedit accepted manuscripts, so the language in submitted articles must be clear, correct, and unambiguous. Any typographical or grammatical errors should be corrected at revision, so please note any specific errors here.

Reviewer #1: Yes

Reviewer #2: Yes

Reviewer #3: Yes

5. Review Comments to the Author

Please use the space provided to explain your answers to the questions above. You may also include additional comments for the author, including concerns about dual publication, research ethics, or publication ethics. (Please upload your review as an attachment if it exceeds 20,000 characters)

Reviewer #1: This study employs a mixed - methods design to explore the factors influencing healthcare - seeking decisions among infertile couples in Islamabad, Pakistan. Combining in - depth interviews and a cross - sectional survey, the research reveals significant socio - psychological pressures and economic and cultural barriers faced by these couples. The results indicate that education level, family structure, and type of infertility significantly impact their decision - making. The study highlights the need for community - based education programs, psychological counseling, and social health protection to improve healthcare - seeking behavior and quality of life for infertile couples. I have some suggestions for future improvement.

1. When discussing the cultural background of Pakistan, the text mentions “Additionally, in developing Asian countries, infertility is often seen as an act of God, a punishment for past sins, or the result of witchcraft”. Although this view is mentioned, the paper could further explore how this cultural perception specifically affects the healthcare - seeking decisions of infertile couples. For example, it could add an analysis of how this cultural belief leads to delays or resistance to medical intervention and how this belief varies among different social classes or educational levels.

Response: Thank you so much we have incorporated this in the introduction section

These cultural beliefs significantly influence healthcare-seeking decisions among infertile couples. Beliefs in evil forces and supernatural powers as causes of infertility remain prevalent, particularly among populations with lower educational levels, leading couples to seek treatment from faith healers before consulting medical professionals (9, 10). Research from Pakistan demonstrates that 35.2%-44.2% of individuals consider spiritual healers as a primary treatment option for infertility (11). This perception varies notably across educational levels and socioeconomic strata. Studies from India reveal that couples with higher education (more than 10 years of schooling) are three times more likely to seek allopathic treatment compared to those with no formal education (10, 12). Conversely, couples with lower education levels may delay medical consultation by 1-3 years, instead pursuing spiritual or traditional solutions (12, 13). Economic factors compound this issue, as traditional remedies are often perceived as more affordable than medical treatment, creating a complex interplay between cultural beliefs and financial constraints that shapes treatment-seeking timelines (14, 15). In South Asian contexts, where infertility is frequently attributed to divine punishment or ancestral curses, couples often prioritize religious rituals and traditional sacrifices over biomedical interventions, particularly in rural areas where healthcare literacy remains limited (7, 16).

2. The text states “The study population comprised married couples in Islamabad who had unsuccessfully tried to conceive for 12 to 18 months, experiencing primary and secondary infertility (having at least one live child), aged 18–35 years and were recruited from a private hospital providing infertility treatment”. The sample being drawn from only one private hospital in Islamabad may not be representative of infertile couples across different regions and economic levels in Pakistan. The authors may have had to choose a specific sample source due to resource or practical constraints. It is suggested that the limitations of the sample could be further discussed in the discussion section. For example, it could mention how this sample selection might affect the generalizability of the study results. If future studies could consider sampling from different types of medical institutions (such as public and private) and different regions (urban and rural), it would help improve the representativeness of the sample.

Response: we have added this in limittaions section and have added following

The study has several limitations. Firstly, the sample was limited to couples seeking treatment at a single private hospital in Islamabad, which significantly affects the generalizability of findings. This sampling approach introduces several biases: (a) it excludes couples who cannot afford private healthcare and rely solely on public facilities, where services may be limited or unavailable; (b) it does not capture the experiences of couples in rural areas who face geographic barriers to specialized infertility care; (c) private hospital patients likely represent a more economically advantaged subset with higher health literacy and greater agency in decision-making. Consequently, our findings may underestimate the barriers faced by lower-income couples and overrepresent treatment-seeking behaviors among more educated, urban populations. Future research should employ multi-site sampling strategies encompassing both public and private facilities across urban and rural settings to enhance representativeness and capture the full spectrum of healthcare-seeking experiences among infertile couples in Pakistan.

Additionally, our facility-based sampling excludes couples who have not yet sought medical care due to financial constraints, stigma, lack of awareness, or geographic isolation. A community-based study design would provide more comprehensive insights into barriers preventing initial healthcare contact and capture the full spectrum of infertility experiences, including those who rely exclusively on traditional or alternative remedies. Future research should employ community-based approaches, such as household surveys or population-based cohort studies, to identify and characterize infertile couples outside healthcare

Furthermore, our study did not collect detailed data on biological, clinical, or genetic factors contributing to infertility (e.g., specific diagnoses such as polycystic ovary syndrome, endometriosis, male factor infertility, or chromosomal abnormalities). We also did not separately analyze risk factors and healthcare-seeking patterns for male versus female factor infertility. This represents a significant gap, as the etiology of infertility and gender-specific attribution may substantially influence treatment-seeking decisions, stigma experiences, and emotional burden. Future studies should incorporate comprehensive clinical assessments, differentiate male and female factor causes, and examine how diagnostic outcomes influence subsequent healthcare-seeking behaviors and psychological wellbeing.

3. When presenting the results of the multivariate logistic regression analysis, “Regression analysis shows that individuals with primary or lower education are 0.2 times less likely to seek healthcare than those with middle or higher education”. Although the impact of education level on healthcare - seeking decisions is given, the underlying mechanisms of this impact could be further explored. For example, it could analyze, in combination with relevant literature, whether people with lower education levels have insufficient knowledge about infertility treatment or whether they face more economic pressure or other social factors.

Response: Thank you for this we have made the changes and added following in discussion section

The strong association between education level and healthcare-seeking behavior identified in our regression analysis (OR=0.260, p<0.001) warrants deeper examination of underlying mechanisms. Lower educational attainment influences treatment-seeking through multiple pathways: (i) **Knowledge gaps**: individuals with limited education often lack understanding of reproductive biology, fertility windows, and available treatment options, leading to delayed recognition of infertility as a medical condition requiring intervention; (ii) **Health literacy barriers**: difficulty navigating complex healthcare systems, understanding medical terminology, and interpreting treatment options creates additional obstacles; (iii) **Economic constraints**: lower education typically correlates with reduced earning capacity, making costly infertility treatments financially prohibitive; (iv) **Social factors**: less educated individuals may have stronger adherence to traditional beliefs about infertility causation (divine punishment, witchcraft) and greater reliance on alternative medicine or spiritual interventions. Furthermore, lower education often intersects with limited social capital and reduced access to information networks that could facilitate appropriate healthcare-seeking. These findings align with studies from India and Rwanda showing that literacy and awareness significantly impact treatment initiation timelines (12, 22).

4. In the conclusion section, it mentions “Recognising infertility as an important public health issue, it is crucial to develop policies that ensure accessible, equitable, and supportive reproductive healthcare for everyone”. Although the general direction of policy recommendations is given, more specific policy measures could be proposed in response to the specific problems found in the study, such as social stigma and economic burden. For example, it could suggest how policies could be used to change the social stigmatization of infertility through publicity or how the government could formulate subsidy policies to alleviate the economic burden on low - income families.

**Response:** we have made the suggested changes and we have added following in conclusion and way froward section

Based on our findings, we recommend the following specific policy interventions:

Launch nationwide public awareness campaigns through mass media, mosques, and community centers to normalize infertility as a medical condition rather than a source of shame. Integrate reproductive health education into school curricula and pre-marital counseling programs. Establish peer support groups and online forums for infertile couples to reduce isolation. Train religious leaders and community influencers to deliver accurate, destigmatizing messages about infertility.

Expand the Sehat Sahulat Program, the national health insurance, to include comprehensive infertility diagnostics and treatment coverage. Establish subsidized fertility clinics in public hospitals with sliding-scale fees based on family income. Mandate private insurance plans to cover at least basic infertility investigations and first-line treatments. Additionally, create government-subsidized loan programs with flexible repayment terms to assist with advanced reproductive technologies.

Establish infertility specialty units in public tertiary hospitals across all provinces. Train general practitioners and gynecologists in basic infertility management to provide care in underserved areas. Develop telemedicine consultation services for couples in rural or remote locations. Create referral pathways from primary care to specialized fertility centers.

Mandate psychological counseling as a standard part of all infertility treatment protocols and train healthcare providers in trauma-informed, culturally sensitive counseling techniques. Establish dedicated mental health services within fertility clinics and develop culturally adapted support materials in Urdu and regional languages.

Reviewer #2: It would have been better if the study was conducted at community level to include all infertile couples who have not visited health care settings yet. The way the data were analyzed lacks some clarity and details. Biological, clinical and genetic factors influencing infertility were not included in the study. Risk factors for males and females were not separately identified (described).

Response: We have incorporated this suggestion as part of limitation

Additionally, our facility-based sampling excludes couples who have not yet sought medical care due to financial constraints, stigma, lack of awareness, or geographic isolation. A community-based study design would provide more comprehensive insights into barriers preventing initial healthcare contact and capture the full spectrum of infertility experiences, including those who rely exclusively on traditional or alternative remedies. Future research should employ community-based approaches, such as household surveys or population-based cohort studies, to identify and characterize infertile couples outside healthcare

Furthermore, our study did not collect detailed data on biological, clinical, or genetic factors contributing to infertility (e.g., specific diagnoses such as polycystic ovary syndrome, endometriosis, male factor infertility, or chromosomal abnormalities). We also did not separately analyze risk factors and healthcare-seeking patterns for male versus female factor infertility. This represents a significant gap, as the etiology of infertility and gender-specific attribution may substantially influence treatment-seeking decisions, stigma experiences, and emotional burden. Future studies should incorporate comprehensive clinical assessments, differentiate male and female factor causes, and examine how diagnostic outcomes influence subsequent healthcare-seeking behaviors and psychological wellbeing.

Reviewer #3: Congratulations to the authors on the completion of the study and on putting together a good report on a relevant subject.

However, I have a few comments.
s/n Section Comments Recommendations

1 Data Collection, Management and Analysis Sampling methods seem unclear; was it systematic random as stated here or purposive sampling as stated in the limitations Consider reviewing and harmonizing these.

Response: Thank you for the feedback we have made following changes in the study design section

The sample size was calculated using OpenEpi, based on a 22% prevalence rate of infertility in Pakistan (8), which yielded a required sample of 268 couples at a 95% confidence interval with a 5% margin of error. Recruitment followed strict inclusion and exclusion criteria to ensure relevance. For the qualitative component, twelve in-depth interviews were conducted with women facing primary and secondary infertility using purposive sampling to capture diverse experiences; recruitment continued until thematic saturation was achieved.

2. There is a repetition of the inclusion criteria in the last paragraph with additional information about use of contraception that was not part of the initial list. Consider harmonizing these and avoid repetitions

Response: We have removed this and rephrased

- Couples/women who were facing primary infertility and have failed to conceive for ≥12 months or more
- Couples/women facing secondary infertility and have not been able to conceive for ≥12 months or more
- <span style="background-color: yellow;">Neither partner currently using contraception</span>
- Not currently pregnant or lactating
- Seeking or having sought infertility treatment
- All those who consented to participate in the research study were interviewed

3 Quantitative findings The second sentence seeks to report the “average age” but a range was stated. Kindly state the mean (with SD).

Response: We have changed and added ‘The mean age of participants was 28.4 years (SD=4.7), with 168 (62.5%) falling within the 20-34 years age range’

4 The 5th sentence (Most had been married…) is unclear and has a mathematical error; 139 of 269 is not 44.6%. Consider recalculating and rewording this.

Response: We have reworded ‘Most had been married for 1-5 years (n=130, 48.3%), and 120 (44.6%) had been attempting to conceive for the past 1-3 years’.

5 Table 1 Under the ‘Treatment advised by’, what does ‘None’ represent? Missing data? Considering self-seeking is a category here. Kindly provide clarifications.

Response: Following added as note

*None refers to participants who sought treatment without any external advice or recommendation from family, friends, or household members. These individuals made independent decisions to seek care without consultation*

6 Table 2 It is unclear how you have two categories of ‘Decision to seek medical care for infertility’ as Yes and No. Giving 100% of the participants already sought medical care.
More so, one cannot trace where the 175 participants under ‘Yes’ had come from. Kindly review and provide clarifications.

Response: We have corrected this. The table expalins what infleunced them to take the decion for seeking infertility treatmnet so table 2 is ‘Association of factors influencing in making decision to seek medical care for infertility’

7 Similarly, there are 81 participants who family and friends advised on table 1 but a total of 87 on table 2 Kindly review and rework

Response: Now corrected in table 1

8  Discussion Factors not adequately discussed Authors should do more in highlighting the factors identified from the study and discussing the logistic regression findings after due diligence with the analyses.

Response: we have explained further in dicussion and have added following

Our logistic regression analysis identified three significant independent predictors of healthcare-seeking behavior for infertility treatment, providing crucial insights for intervention design.

**Educational Attainment**: The strongest predictor was education level (OR=0.260, 95% CI: 0.135-1.498, p<0.001). Individuals with primary or lower education were 74% less likely to seek healthcare compared to those with middle or higher education. This finding aligns with existing literature from India and Rwanda (12, 22) demonstrating that education enhances health literacy, reduces stigma, and facilitates navigation of healthcare systems. Educational interventions targeting low-literacy populations through community health workers and visual communication tools could substantially improve care-seeking rates.

**Family Structure**: Living in a joint family system significantly increased the likelihood of seeking treatment (OR=0.354, 95% CI: 0.183-1.685, p=0.002). While counterintuitive given increased family pressure described in qualitative findings, this likely reflects financial pooling and collective decision-making in joint families. Extended family members may contribute financially to treatment costs and provide emotional support, offsetting the negative impacts of stigma and pressure. However, this also suggests that couples in nuclear families face disproportionate barriers and may benefit from community support structures and financial assistance programs.

**Type of Infertility**: Couples experiencing primary infertility were significantly more likely to seek care (OR=3.254, 95% CI: 0.546-6.850, p=0.002) compared to those with secondary infertility. This finding reflects greater urgency and social pressure for childless couples. Secondary infertility may be perceived as less severe, particularly if couples already have male children, leading to delayed or foregone treatment. Healthcare messaging should emphasize that secondary infertility warrants equal medical attention and that family completion is a legitimate reproductive health goal.

9 The sentences after the citation 29 and 30 seem unfit for that section. Consider moving those to the conclusion.

Response:already addressed in Reviewer #1, Comment 4 above

10 General comment The use of medical ‘advice’ and medical ‘treatment’ interchangeably These two are not technically the same and authors should review and decide which they are referring to.

Response: We have corrected this thank you so much
