# Supplementary material for: Factors influencing infertile couples’ decisions to seek healthcare: A mixed methods study from Islamabad, Pakistan
Source: PLOS Glob Public Health. 2026 Apr 15;6(4):e0006275. doi: 10.1371/journal.pgph.0006275 (PMC13082636; doi:10.1371/journal.pgph.0006275)
Supplement: Attachment — Submitted filename: Response to comments_Infertility .docx [file pgph.0006275.s006.docx]

**Response to Reviewers**

Dear Editors,

We sincerely thank the reviewers for their careful reading of our manuscript and for their thoughtful, constructive comments. We have revised the manuscript in full accordance with the feedback received. All changes are shown as tracked revisions in the accompanying revised manuscript. Our point-by-point responses are provided below.

We are confident the manuscript has been substantially strengthened by this process.

1. **Reviewer #4: This is a well written manuscript, great flow. An important public health concern in many settings. The authors demonstrated the similarity of the issue in most settings across Asia and Africa where cultural beliefs have a great influence on health-seeking behavior and social norms.
   It could be easier for the reviewer to comment and for the author to track comments if the manuscript lines are numbered**

Response: Thank you for this practical suggestion. Line numbers have been added to the revised manuscript to facilitate ease of review and cross-referencing during the revision process.

2. **When authors determine the required sample size, it is appropriate to report the actual size used if it differs from the required**

Response: We appreciate this point. The required sample size was 268 couples, calculated using OpenEpi at a 95% confidence interval with a 5% margin of error, based on a 22% prevalence rate of infertility in Pakistan. The actual number enrolled was 269 participants, reflecting our consecutive sampling approach whereby one additional eligible participant presented during the study period. This has been clarified in the Methods section.

3. **The description of the qualitative analysis is insufficient. The authors state that thematic content analysis was conducted manually, but additional details are needed regarding the analytic approach, coding procedures, number of coders, development of the codebook, consensus procedures, saturation assessment.**

Response: We fully agree with this observation and thank the reviewer for identifying this gap. The Methods section has been expanded to specify the following: (i) two researchers independently coded transcripts using an inductive approach; (ii) initial codes were generated line-by-line and progressively organised into themes and subthemes through iterative comparison across transcripts; (iii) a codebook was developed collaboratively and refined as analysis progressed; (iv) discrepancies between coders were resolved through discussion and consensus; and (v) data saturation was assessed throughout data collection and confirmed when no new themes emerged — which occurred at the twelfth interview. Please see the tracked change in the Data Collection, Management and Analysis section Line number 207

4. **“Data triangulation was employed to ensure validity and reliability”. Kindly give details on this process.**

Response: Accepted. The revised text now specifies that triangulation involved cross-verification of findings across three sources: quantitative survey data, qualitative interview transcripts, and relevant published literature. The changes have been made in the manuscriot Line 223 onwards .

5. **In the results table 1 Factors influencing the decision to seek treatment, the authors reported 4 items, one of which is unable to conceive, which is the definition of infertility. This mimics the title of the table, not results, as the other 3 items are all describing the reason to seek treatment for the inability to conceive. This item doesn’t give accurate information.**

Response: This is a perceptive observation and we are grateful for it. You are correct that "unable to conceive" effectively restates the clinical condition rather than capturing a meaningful motivating factor distinct from the others in the table. We have revised this label to "Desire for parenthood (self-motivated concern)" to more accurately reflect the internal motivation, the wish to have a child, that drove participants to seek care, distinguishing it from the three externally driven factors. The corresponding text in the Results section has been updated accordingly. The tracked changes are visible in both the table and the results narrative.

6. **In the discussion, the association between social factors and lower educational attainment may not consistently predict health behaviors. Highly educated and well-informed individuals often adhere strongly to generational beliefs and cultural norms that influence health-related perceptions and opinions**

Response: This is an important point that we had not adequately addressed in the original manuscript. The revised Discussion now explicitly acknowledges that educational attainment does not uniformly predict health-seeking behaviour, and that in settings such as Pakistan, highly educated individuals may continue to hold and act upon deeply ingrained generational and cultural beliefs around infertility, including perceptions of divine will, family honour, and social duty. Education may reduce certain knowledge barriers while leaving cultural and normative influences largely intact. We note that this nuance should be borne in mind when designing education-based interventions, which ought to address cultural framing alongside information provision. The tracked change is in the Discussion section under the heading on educational attainment (line 362-369)

**_________________________________________________________________________________**

**Reviewer #5:**

1. **The study would benefit from data from study location on the infertility rates and then presented in the whole Country perspective.**

Response: We appreciate this suggestion. Unfortunately, city-level or district-level data on infertility rates specific to Islamabad are not currently available in the published literature. The Introduction already presents the national figure (approximately 22% female infertility rate in Pakistan). We have added a sentence acknowledging the absence of disaggregated local-level data and flagging this as a gap in the existing evidence base. Future population-based studies with geographic disaggregation would help address this need.

2. **In the research questions, not sure why the first 2 questions were asked on the couples and the third yet important one only on women? Do we have findings that support that challenges are faced more or only by women? As for mixed methods study, as quantitative surveys were filled by couples and women who ere seeking treatment in one private healthcare facility, with qualitative interviews, why only woman were sampled? It is imperative that couples are sampled in the design to justify the research objective.**

Response: This is a fair and important concern. The third research objective was specifically designed to explore the lived experiences and challenges faced by women, for reasons grounded in the South Asian literature and in our own quantitative and qualitative findings. Evidence consistently shows that in Pakistan, and more broadly in South Asia, the social and emotional burden of infertility falls disproportionately on women: they are more frequently blamed for the failure to conceive, more commonly subjected to family pressure, social isolation, and psychological distress, and are the primary seekers of infertility treatment. Capturing these experiences required a women-focused qualitative inquiry with open, exploratory interview guidelines.

We fully acknowledge, however, that excluding male partners from the qualitative component limits insight into men's perspectives and experiences, a significant gap, particularly given the mixed-methods design and couple-level framing of the quantitative component. A clarifying sentence has been added to the Methods section to explain this decision, and the limitation has been strengthened by an explicit recommendation for future studies to incorporate qualitative data from both male and female partners. The tracked change is visible in both the Methods line 120-130 and the Limitations sections.

3. **Logistic regression findings on the predictors (educational level, family structure, type of infertility) look like could be chance finding. Would like to get the statistical teams comment on this.**

Response: We thank the reviewer for raising this point. The logistic regression was built using a conservative screening threshold (p < 0.20 at univariate level) and model fit was confirmed using the Hosmer-Lemeshow test (p = 0.80). However, we agree that the confidence intervals for the family structure variable (95% CI: 0.183–1.685) and the primary infertility variable (95% CI: 0.546–6.850) are notably wide, and that these associations should be interpreted with appropriate caution. The revised text now includes an explicit caveat that some associations, while statistically significant, may reflect the relatively modest sample size and single-site design, and that these findings are best regarded as exploratory pending replication in larger, multi-site studies. The tracked change is in the Results section following Table 3.

4. **The recommended policy interventions need feasibility and pragmatic approach. As a suggestion would be good to have Key Informant Interviews (KIIs) with the key stakeholders on this agenda and plan what is appropriate.**

Response: We agree entirely. The Way Forward section has been revised to include a recommendation that, before any of the proposed interventions are taken to scale, Key Informant Interviews (KIIs) be conducted with key stakeholders — including health policymakers, facility administrators, community leaders, and healthcare providers. This stakeholder engagement would allow for a pragmatic assessment of the feasibility of each recommendation, identification of context-specific barriers, and refinement of strategies to ensure they are sustainable and appropriate within the Pakistani health system. The tracked change is visible at the end of the policy recommendations paragraph.

5. **The limitation on the sampling from one private hospital will need more attention as the recommendations made could be affected or might have to be varied based on the sampling location and sampling characteristics. The recommendation to run a community level research exploring male factors will need followup and strategies to make it a reality.**

Response: We appreciate this important critique. The Limitations section already discusses the single-site private hospital constraint in some depth, noting exclusion of public sector patients and rural populations. We have strengthened this section to more explicitly acknowledge that our findings may not be transferable to lower-income groups, public facility users, or geographically dispersed populations — and that recommendations derived from this study should be understood within these constraints.

Regarding the recommendation for community-based research on male factors, we have retained this recommendation in the text but have revised the framing to include concrete strategies that would make such research feasible and meaningful: multi-site sampling spanning public and private facilities, community-based household survey designs, and deliberate inclusion of male partners as equal participants in both quantitative and qualitative components. We believe this more actionable framing addresses the reviewer's concern directly.

6. **As for the study design, the questionnaire used looks like the responses could have been influenced by the nature of the questions. The responses on sensitive areas might not yield valid responses if asked as binary or leading questions. The strong suggestion would be to use qualitative study (KIIs with couples, Focus group discussions (FGDs) with families, community, health care professionals) to get comprehensive narration of the underlying contributing factors and the intrinsic mechanisms that operate and influence the healthcare seeking behaviour of the sample studied.**

Response: This is a valid methodological concern and we appreciate it. The questionnaire was adapted from a validated instrument used in a comparable South Asian context (Patra & Unisa, 2022) and was pilot-tested and refined prior to use, achieving a Cronbach's alpha of 0.85. Nevertheless, we acknowledge that closed-ended survey questions on sensitive topics, such as stigma, blame, and decision-making autonomy — inherently carry risk of social desirability bias, particularly in the Pakistani sociocultural context.

We strongly agree that future research in this area would benefit from a richer qualitative design, including KIIs with couples and healthcare professionals, and FGDs with families and community members, to generate the kind of in-depth narrative insight that surveys alone cannot capture. This recommendation has been added to the conclusion and future research section as a tracked change, and we have flagged the potential for social desirability bias in the Limitations section.

**We hope that the revised manuscript and these responses adequately address the concerns raised. We remain available to provide any further clarification and are grateful for the opportunity to strengthen this work.**

Yours sincerely,

**Dr Mariam Ashraf (Corresponding Author)**
